# CW_ICA: an efficient dimensionality determination method for independent component analysis

**DOI:** 10.1038/s41598-023-49355-z

**Published:** 2024-01-02

**Authors:** Yuyan Yi, Nedret Billor, Arne Ekstrom, Jingyi Zheng

**Affiliations:** 1 Department of Mathematics and Statistics, Auburn University, Auburn, AL 36849 USA; 2 Department of Psychology and Evelyn McKnight Brain Institute, University of Arizona, Tucson, AZ 85721 USA

**Keywords:** Learning algorithms, Data processing, Statistical methods, Statistics

## Abstract

Independent component analysis (ICA) is a widely used blind source separation method for signal pre-processing. The determination of the number of independent components (ICs) is crucial for achieving optimal performance, as an incorrect choice can result in either under-decomposition or over-decomposition. In this study, we propose a robust method to automatically determine the optimal number of ICs, named the column-wise independent component analysis (CW_ICA). CW_ICA divides the mixed signals into two blocks and applies ICA separately to each block. A quantitative measure, derived from the rank-based correlation matrix computed from the ICs of the two blocks, is utilized to determine the optimal number of ICs. The proposed method is validated and compared with the existing determination methods using simulation and scalp EEG data. The results demonstrate that CW_ICA is a reliable and robust approach for determining the optimal number of ICs. It offers computational efficiency and can be seamlessly integrated with different ICA methods.

## Introduction

Independent component analysis is a statistical tool to extract hidden information from the observed (mixed) signals. Assuming that these observed signals are linear combinations of mutually independent and non-Gaussian source signals, ICA seeks to discover the linear combination of these mixed signals to recover the original source signals. The performance of ICA is measured by the independence or non-Gaussianity of the estimated ICs.

ICA finds extensive applications in various disciplines. In the realm of biomedical science, it has been leveraged to investigate the brain function, primarily by extracting temporal and spatial information from functional magnetic resonance imaging (fMRI)^1,2^ and electroencephalograph (EEG)^3^. In pharmaceutical fields, ICA enables the examination of the distribution of actives and major excipients within tablets by comparing the calculated signals with the pure spectra of the formulation compounds^4^. In chemistry, ICA is widely used for the separation of unknown sample mixtures^5^ via peak detection and matching in high-performance liquid chromatography. Additionally, ICA is also extensively used in signal pre-processing for identifying and removing noise and contaminations, thereby extracting effective information.

However, a crucial challenge when utilizing ICA lies in determining the optimal number of ICs for the accurate decomposition. Both under-decomposition (too few ICs) and over-decomposition (too many ICs) can hamper effective source separation. Commonly used determination techniques include information criteria, eigenvalue spectrum (ES), bootstrap resampling (BS), and cross-validation (CV), among others. Nevertheless, these methods have their drawbacks. For instance, information criteria may suffer from overfitting when the sample size is small or strict model assumptions are made. Eigenvalue spectrum methods can be subjective in the choice of threshold and may be affected by noisy signals. Bootstrap resampling techniques, although comprehensive, can be computationally expensive. Cross-validation, while generally reliable, may introduce data partition bias and incur computational costs.

To address the issues with these determination methods, researchers have proposed several alternatives, that can be categorized into three classes. Firstly, methods leveraging original signal information have been introduced. Wang et al.^6^ introduced Mean-field ICA (MF-ICA), a Bayesian-based approach that determines the optimal number of ICs by evaluating the square-root sum of the residual between original and reconstructed data. This method excels in separating complex mixtures, such as those encountered in chemistry. Other approaches in this class, such as those proposed by Monakhova et al.^7^ and Kassouf et al.^8^ leverage different metrics for the determination of the optimal number of ICs. Monakhova et al. introduced an index, known as the Amari Index, whereas Kassouf et al. employed a correlation method, referred to as ICA_corr_y. Both methods, however, necessitate prior knowledge of the ground truth or mixing matrix. Secondly, there are methods employing visual analysis. Bouveresse et al.^9^ proposed two techniques, one of which is the ICA-by-Blocks method, employing a “signal-correlation” plot to determine the optimal number of ICs. The other method uses a heatmap generated by the Durbin–Watson criterion. Thirdly, there are methods that require specific data structures. Bach et al.^10^ suggested a determination method based on a forest-structured graphical model, which is limited to dependencies among sources within a forest structure and may not apply to broader classes of dependencies. Kassouf et al.^8^ presented a determination method using the Kaiser–Meyer–Olkin (KMO) index, a measure indicating the presence of a partial correlation among at least two residual signals. Nonetheless, if there is a small number of ICs, this method encounters a potential pitfall in cases of complete or high correlation among variables, which makes the correlation matrix non-invertible and further poses challenges to the analysis. While these methods offer computational efficiency and require fewer assumptions compared to other techniques, they do have certain drawbacks. For instance, they require enough mixed signals and structured signals (such as sparse, periodic, linear, etc.). Additionally, in some methods, the optimal number of ICs must be visually identified from a plot, which can be subjective. Furthermore, the existing methods for determining the number of ICs may not be universally applicable to all ICA methods, which introduce additional challenges, such as uncertainty and instability. It is also worth noting that the robustness of the determination method for signals with different characteristics plays an important role in determining the optimal number of ICs. However, this factor has not been extensively studied in the context of existing determination methods (see Table 1 for a summary of advantages and disadvantages of these techniques categorized into three groups).

**Table 1:** Summary of existing determination methods.

Category	Selected methods	Pros	Cons
Require original source signals information	MF-ICA	Effective for separating complex mixtures, particularly in Chemistry	Requires original source signals
	Amari index based	A quantitative measure with intuitive interpretation	Requires true mixing matrix
	ICA_corr_y	A data-driven approach with computational efficiency	Requires at least one original source signal
Visual determination	ICA-by-Blocks	Flexible in block size, easy interpretation via plot	High computational complexity
	DW criterion	Determine the number of IC based on signal/noise ratio	Fails if variance of DW values among mixed signals is large
Require specific data structure	FCA	Model both inter-cluster independence and intra-cluster dependence	Requires forest structured signals
	KMO index based	A quantitative measure with intuitive interpretation	Fails for cases where small number of ICs occurs
	DW criterion	Determine the number of IC based on signal/noise ratio	Requires structured signals

Given the limitations of current determination methods, we propose a method called column-wise independent component analysis (CW_ICA) to automatically determine the optimal number of ICs. Inspired by the ICA-by-Blocks approach, the proposed method addresses challenges related to computational efficiency, consistency, and robustness. Instead of using Pearson correlations, CW_ICA employs Spearman correlations among the ICs obtained from the different blocks. This choice offers advantages in terms of capturing monotonic relationships between ICs, which can be valuable in various scenarios. Moreover, we introduce a novel metric based on the column-wise maximum rank-based correlations between the extracted ICs in CW_ICA. This metric serves as a criterion for determining the optimal number of ICs. Therefore, compared to the existing determination methods, the major advantages of CW_ICA are:

1. **Efficiency**: the computational cost is significantly less than existing methods.
2. **Consistency**: the optimal numbers of ICs obtained by CW_ICA are consistent when it is coupled with different ICA methods.
3. **Robustness**: CW_ICA is robust for signals with different characteristics.

The rest of the paper is organized as follows. In section “Background”, we provide a brief introduction to the ICA method and review the current determination methods. Section “Proposed CW_ICA method” presents the CW_ICA method, using a simple example for illustration. We also compare the CW_ICA method with existing determination methods using both simulation and real data in order to evaluate its performance in section “Assessment of proposed method”. A detailed discussion of our findings can be found in section “Discussion”, and our conclusion is outlined in last section.

## Background

### Fundamentals of ICA

Suppose there are *p* mixed signals with length being *n*. Denote the observed signal matrix as ${\varvec{X}}_{p \times n}$, where *p* is the number of mixed signals and *n* is the signal length. Assuming the observed signals are linear mixtures of *q* source signals. Then the ICA model is formulated as:

1 $$ {\varvec{X}}_{p \times n} = {\varvec{A}}_{p \times q} {\varvec{S}}_{q \times n} $$

where ${\varvec{A}}_{p \times q}$ is the “mixing matrix”, which specifies the contributions of the source signals to each mixture, and ${\varvec{S}}_{q \times n}$ is the matrix of source signals. ICA aims to determine both the mixing matrix and the source signal matrix. According to the number of observed signals (*p*) and source signals (*q*), ICA methods can be divided into two cases: (over)determined ICA (i.e., $p \ge q$) (e.g., FastICA^11^, JADE^12^, Infomax^13^, etc.), and underdetermined ICA ($p < q$) (e.g., FastFCA^14^, MAICA^15^, OICD^16^, etc.). In this paper, we focus on (over)determined ICA ($p \ge q$), where the mixing matrix ${\varvec{A}}$ is invertible. The object of ICA can be achieved by estimating the de-mixing matrix ${\varvec{W}} = {\varvec{A}}^{ - 1}$, and the estimated source signals (ICs) can be further obtained by projecting the whitened data onto the matrix ${\varvec{W}}$.

For comparison purposes, we employed the three most commonly used ICA methods—FastICA, Infomax and JADE when combined with the determination methods.

### Overview of existing determination methods

The selection of determination methods is contingent upon the specific structure and characteristics of the data under consideration. In this section, we provide a concise summary of the current state-of-the-art in this field, highlighting key differences among these methods. For a detailed comparison of the pros and cons of the existing determination methods, please refer to Table 1.

Outlined below are algorithms for three different determination methods. These methods will be compared with the proposed technique in “Proposed CW_ICA method”. In the algorithms provided, ${\varvec{X}}_{p \times n}$ represents $p$ mixed signals each of length $n$. The residual signal matrix is calculated by subtracting estimated signals from initial signals, ${\varvec{X}}$, denoted by ${\varvec{R}} = {\varvec{X}} - \hat{\user2{X}}$. $A_{max}$ represents the maximum number of ICs.

#### Durbin–Watson (DW) criterion

The DW statistic is a well-known test statistic used for detecting the presence of autocorrelation in the residuals from a regression analysis^17^. It also serves as a measure of the signal-to-noise ratio in signals, which provides a method for determining the number of ICs^18^. The value of DW criterion for the *i*th mixed signal, $X_{i}$, is defined as:

2 $$ DW_{i} = \frac{{\mathop \sum \nolimits_{t = 2}^{n} (r_{i,t} - r_{i,t - 1} )^{2} }}{{\mathop \sum \nolimits_{t = 1}^{n} (r_{i,t} )^{2} }}, \quad i = 1, \ldots ,p $$

where $r_{i,t}$ denotes the value of *i*th residual signal at time point *t*. If the *DW*_*i*_ is close to 0, the signal is noise-free, implying that the extracted IC necessitates further decomposition, However, if it is near 2, the signal is inundated with noise, which indicates that the signal is over-decomposed. The average of *DW*_*i*_ values over all *p* signals is employed as a measurement for determining the number of ICs. Nevertheless, the variance of* DW*_*i*_’s tends to be large in real datasets due to the non-linear behavior exhibited in real-world signals, which contradicts the linearity assumption inherent to ICA methods. In practice, heatmaps are used to depict DW values for each mixed signal produced by models with varying numbers of ICs. From the heatmap plot, *q*_*opt*_ is determined to be the optimal number of ICs whenever a sudden increase occurs in the DW values of all mixed signals. The procedure that determines the optimal number of ICs based on the heatmap is summarized as below:

**Figure:**
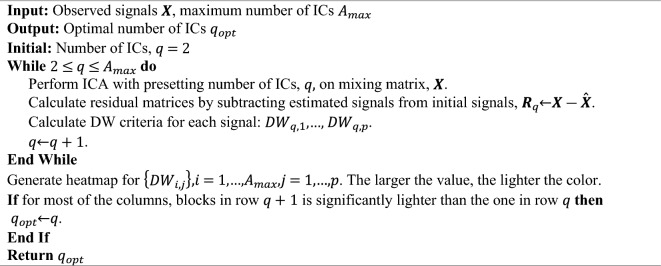
Algorithm based on the Durbin–Watson criterion.

Due to the large variance of DW values among mixed signals, especially for the large number of mixed signals, it becomes challenging to visually select the optimal number of ICs. Further, another limitation of Durbin–Watson criterion is that it can only be used for structured signals (i.e., sparse, periodic, linear, etc.).

#### ICA_corr_y

ICA_corr_y was proposed to select the optimal number of ICs but requires a known source signal (*y*)^8^. The main focus of this method lies in determining the correlation between the estimated source signals ($\hat{\user2{S}}$) and the known source signal (*y*). The highest correlation is expected to be observed when the optimal IC number is extracted, despite potential experimental errors. In other words, among ICA models with different number of ICs, if the ICA model with *q*_*opt*_ ICs includes the IC with the highest correlation to the known signal *y*, *q*_*opt*_ is identified as the optimal number of ICs. Details of the algorithm is presented below:

**Figure:**
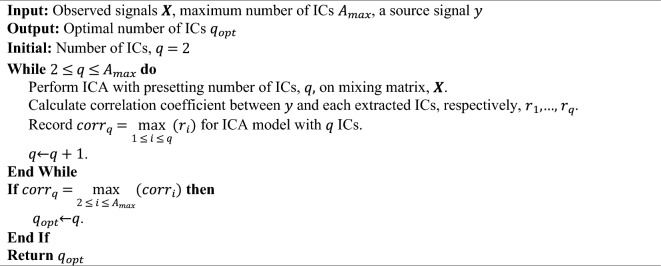
Algorithm ICA_corr_y.

However, having at least one source signal known is a strong requirement, and is not common in many scientific fields such as the scalp EEG signals. Due to this stringent requirement, despite its simplicity, this method is not widely adopted.

#### ICA-by-blocks

ICA-by-Blocks was proposed to determine the number of ICs based on the correlation of ICs between blocks^9^. The original data matrix is split into *B* blocks, which is decided in advance. Then, *A*_*max*_ ICA models with 1 to *A*_*max*_ ICs are computed for each of these predefined blocks. ICs corresponding to “true” source signals are expected to be found in all blocks. Such “true” ICs derived from different blocks should be highly correlated with each other. If all extracted ICs in each block are “true” ICs, the correlation between these ICs in different blocks should be close to 1. If too many ICs are extracted from the blocks, the extraneous ICs will contain a substantial portion related to noise, causing them to exhibit markedly lower correlations with all the ICs derived from other blocks. The ICA-by-Blocks algorithm is outlined below:

**Figure:**
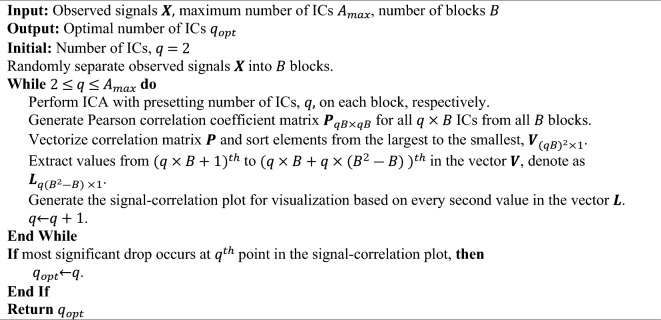
Algorithm ICA-by-blocks.

However, this method is constrained by the number of mixed signals, *p*, since both the choice of the number of blocks, *B,* and maximum number of ICs, *A*_*max*_, depend on the sample size (i.e., $A_{max} \le \frac{p}{B}$). While multiple blocks are desired to better measure the correlation between ICs extracted from different blocks, too many blocks will restrict the maximum number of ICs and increase the computational complexity. Further, this method does not use a quantitative measure to determine the optimal number of ICs, instead a signal-correlation plot is used visually to determine it. Thus, it is rather time-consuming and potentially prone to subjective errors.

## Proposed CW_ICA method

### Algorithm development

**Figure 1:**
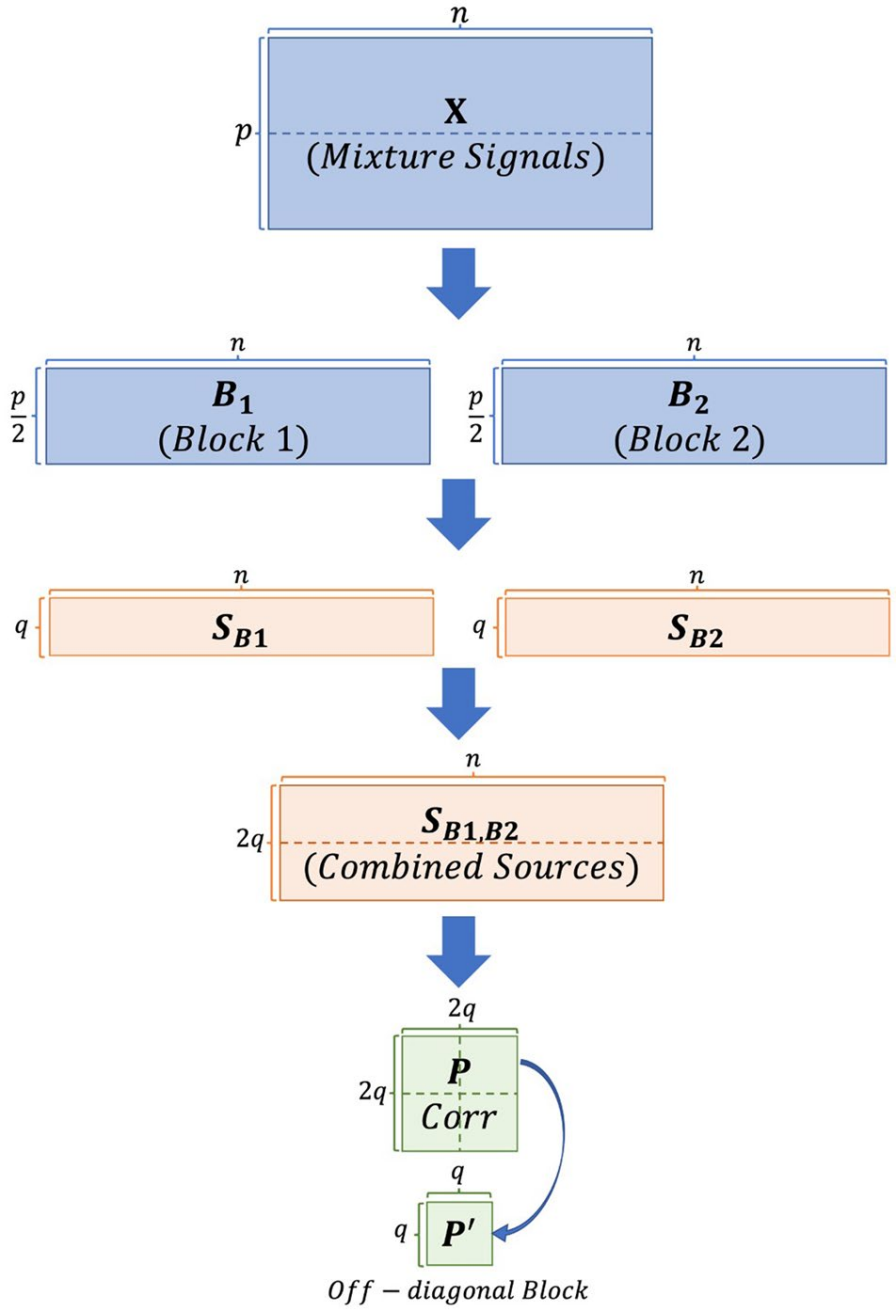
Stages of data structures in CW_ICA method.

The CW_ICA method starts by randomly splitting the initial data matrix into two sample blocks (${\varvec{B}}_{1}$ and ${\varvec{B}}_{2}$), each containing an approximately equal number of signals. The maximum number of computed ICs, denoted by *A*_*max*_, is preset and assumed to be less than the number of signals in each block, ($\frac{p}{2}$). When $p$ is an odd number, the mixed signals are randomly divided into two blocks, with one containing $\frac{p + 1}{2}$ signals and the other containing $\frac{p - 1}{2}$ signals. We then perform ICA models on each block respectively with the same number of ICs. The process is repeated with the number of ICs varying from 2 to *A*_*max*_. ICs corresponding to the true source signals are expected to be in two blocks. We assume that for each true IC extracted from Block 1, there is a highly correlated IC extracted from the Block 2. In order to measure the correlation between ICs from different blocks, we use a rank-based correlation matrix,${\mathbf{\rm P}}_{2q \times 2q}$, that measures a monotonic relationship among extracted ICs, in other words, not restricted to linear relationship as the Pearson correlation matrix does. Further, due to the symmetry of the correlation matrix, we only need to perform further analysis on the off-diagonal block, ${\mathbf{{P}^{\prime}}}_{\;q \times q}$ (see Fig. 1 for a detailed process).

For each model with *q* ICs, we record the maximum absolute value of each column in ${\mathbf{{P}^{\prime}}}$, indicating the strongest correlation between each pair of ICs ($\rho_{1} ,\;\rho_{2} , \ldots ,\;\rho_{q}$). Then, we record the smallest of these *q* values to represent the least absolute correlation coefficient between a pair of ICs. This leads to a quantitative measurement for the ICA model with *q* ICs, defined as

3 $$ R_{q} = { }\mathop {\min }\limits_{1 \le i \le q} \left\{ {\max \left\{ {|\rho_{i} |} \right\}} \right\} $$

where *r*_*i*_ is the *i*th column of the matrix ${\mathbf{P^{\prime}}}$. When *q* is small, it is likely to underestimate the source signals, resulting in highly correlated extracted ICs, i.e., *R*_*q*_ is closed to 1. As *q* increases, it tends to overestimate, introducing noise signals and causing some extracted ICs to be uncorrelated, i.e., *R*_*q*_ is closed to 0. Therefore, to identify the optimal number of IC, we observe the changes of *R*_*q*_ as *q* grows. The *q*_*opt*_ is selected based on the pattern of* R*_*q*_, where *R*_*q*_ is relatively high while $R_{q + 1}$ is significantly lower. Specifically, when the number of ICs exceeds *q*_*opt*_, if *R*_*q*_ decreases significantly and remains consistently low as *q* increases, we claim that the optimal number of ICs is *q*_*opt*_.

To quantify the “significant drop”, we calculate the first-order difference of $R_{2} ,\; \ldots ,\;R_{{A_{max} }}$

4 $$ D_{i,q} = R_{i,q} - R_{i,q - 1} ,{ }\;\;q = 3, \ldots ,A_{max} ,{ }\;\;i = 1, \ldots ,Rep $$

where $R_{i,q}$ is the smallest column-wise maximum absolute correlation value, $Rep$ is the number of repetitions. A negative value of $D_{i,q}$ signifies a decrease in the measurement, with a smaller value indicating a more substantial decrease. Thus, the optimal number of ICs is automatically selected out according to the index of the smallest first-order difference, that is $\mathop {\min }\nolimits_{{3 \le {\text{q}} \le A_{\max } }} \left\{ {D_{i,q} } \right\}$.

We repeat this procedure multiple times, record the detected optimal number of ICs each iteration. The optimal number of ICs is the one that occurs most frequently over all repetitions. The steps are summarized below:

**Figure:**
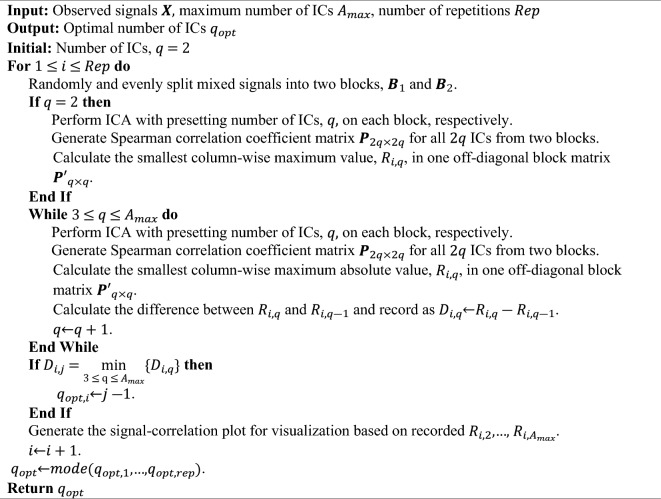
Algorithm CW_ICA.

**Figure 2:**
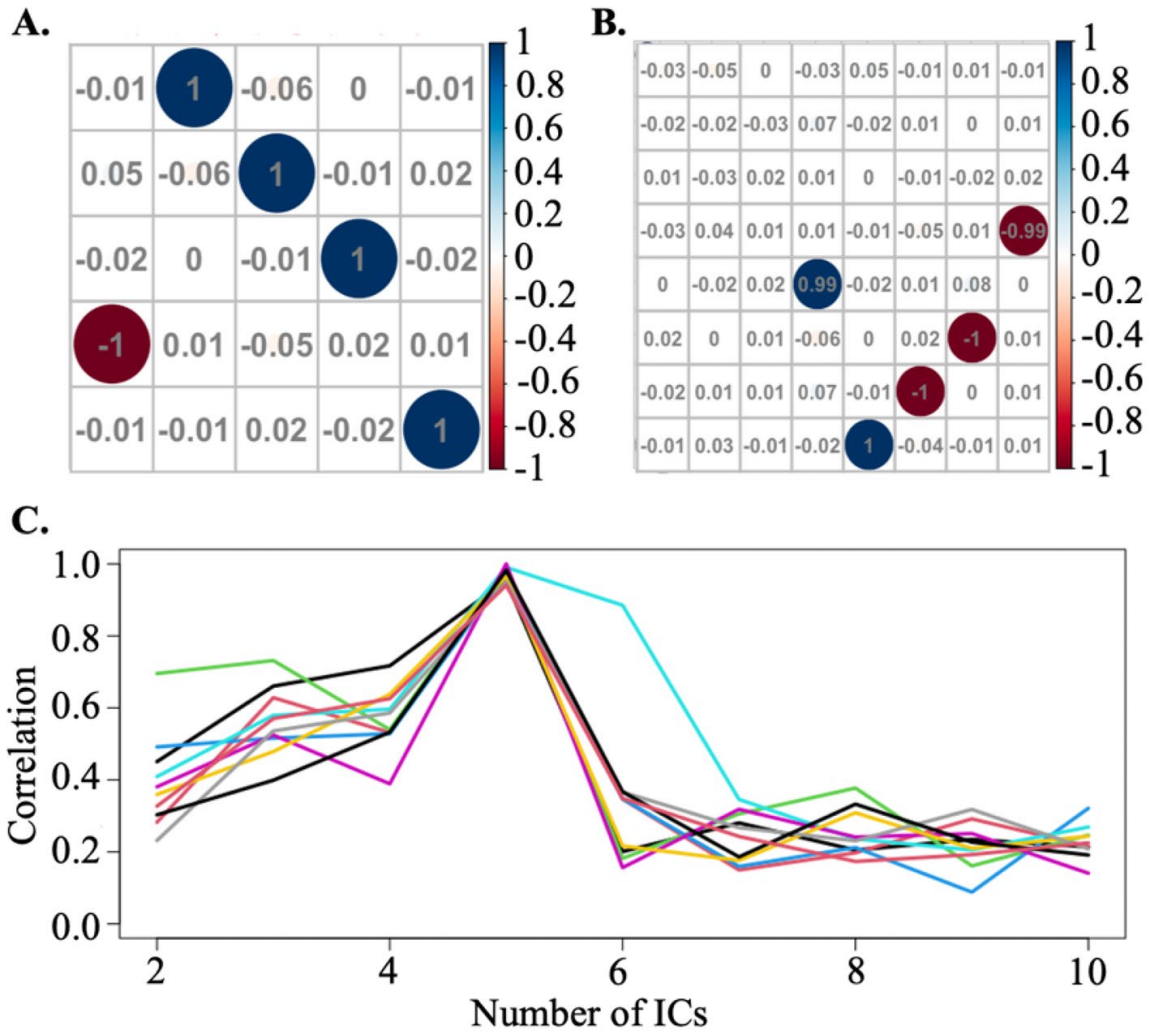
(**A**) Off-diagonal correlation plot for estimated ICs from two blocks (q = 5). (**B**) Off-diagonal correlation plot for estimated ICs from two blocks (q = 8). (**C**) Signal-correlation plot of one replicate. The significant drop at 5th indicates the optimal number of ICs is 5 at this replicate.

The signal-correlation plot shown in Fig. 2C, which depicts the change in $$R_{q} $$ as $$q$$ increases, can also be used to visually identify the optimal number of ICs via detecting a significant drop. However, this method could be time-consuming and may introduce subjective bias.

### Validation

CW_ICA starts by randomly partitioning mixed signals into two blocks. This is pivotal as excessively dividing the data into numerous blocks might result in each block containing only a limited number of ICs^8^. In this method, we suggest using a rank-based correlation coefficient, specifically, the Spearman correlation coefficient, as a measure for determining the correlation between ICs. The Spearman correlation coefficient is a statistical measure used to assess the strength and direction of the relationship (i.e., the monotonic relationship) between two variables. As a nonparametric measure, the Spearman correlation coefficient refrains from making assumptions about data distribution or homoscedasticity. Instead of relying on the actual values, the Spearman correlation is based on ranking the data points for both variables. Moreover, the Spearman correlation is robust against outliers because it relies on ranking rather than actual values, making it less susceptible to the influence of extreme data points. The determination of the optimal number of ICs in the ICA-by-Blocks method heavily relies on visual interpretation of a signal-correlation plot. However, this approach is inherently subjective and potentially time-consuming, particularly when the plot shows multiple significant drops at similar levels. In contrast, the proposed CW_ICA method automates this process by quantitatively defining what constitutes a “significant drop”, thereby eliminating the need for manual interpretation or inspection.

Additionally, the ICA-by-Blocks method lacks repetitions, which increases the risk of obtaining results by chance. To avoid the risk of biased random splitting due to specific row distribution, we propose repeating the entire process—randomization, equitable division of mixed signals into two blocks, and the subsequent steps—multiple times, denoted as *Rep*. This systematic repetition within the CW_ICA methodology ensures a more robust and reliable determination of the optimal number of ICs.

### Illustrative example

To provide a clear explanation of the CW_ICA procedure, we consider a dataset comprising $p = 20$ mixed signals, each with a length of $n = 500$, which are generated by linear combination of 5 source signals ($q_{opt} = 5$). Our objective is to determine the optimal number of ICs, $q_{opt}$.

CW_ICA starts with randomly dividing the 20 mixed signals into two blocks: ${\varvec{B}}_{1}$ and ${\varvec{B}}_{2}$, with the dimension of each block being $\frac{p}{2} \times n\; \left( {{\text{i.e}}{., }\;10 \times 500} \right)$. With the maximum number of ICs being 10 ($A_{max} = 10$), we perform ICA on each block using a range of preset IC numbers $\left( {q = 2, \ldots ,10} \right)$. For instance, when $q = 8$, after applying ICA to both ${\varvec{B}}_{1}$ and ${\varvec{B}}_{2}$, we obtain two sets of ICs, one from each block, resulting in a combined matrix of $2q \times n \;\left( {{\text{i.e}}{., }\;16 \times 500} \right)$. Then we compute the Spearman correlation coefficient between each pair of ICs and the correlation matrix is denoted as ${\varvec{P}}$ with dimension being $2q \times 2q \;\left( {{\text{i.e}}{.,}\; 16 \times 16} \right)$. ${\varvec{P}}$ is composed of four distinct blocks with each block being a $q \times q\; \left( {{\text{i.e}}{.,}\; 8 \times 8} \right)$ matrix. The diagonal blocks are indeed identity matrices because they are the correlation between ICs from the same block, which are orthogonal. The two symmetric off-diagonal blocks, which depict correlations between the ICs in ${\varvec{B}}_{1}$ and ${\varvec{B}}_{2}$, contains the same information. therefore, we only need to consider one off-diagonal matrix, $\user2{P^{\prime}}$, as shown in Fig. 2B,

For each column of $$\user2{P^{\prime}}$$, we first record the highest absolute correlation value ($$\rho_{1} ,\;\rho_{2} , \ldots ,\;\rho_{8}$$), which indicates the strength of correlation between each IC in $${\varvec{B}}_{1}$$ and each IC in $${\varvec{B}}_{2}$$. Then, the quantitative measurement of this model is $$R_{8} = \mathop {\min }\nolimits_{{1 \le {\text{i}} \le 8}} \left\{ {\rho_{i} } \right\}$$. If $$R_{8}$$ is close to 1, it suggests that all extracted ICs in $${\varvec{B}}_{1}$$ have strong correlations with ICs from $${\varvec{B}}_{2}$$. This indicates that the optimal number of ICs is greater than or equal to 8. Conversely, if $$R_{8}$$ is close to 0, it implies that some extracted ICs from $${\varvec{B}}_{1}$$ are not correlated with any ICs from $${\varvec{B}}_{2}$$ These redundant ICs indicate that the optimal number of ICs should be less than 8. In Fig. 2B, we observe that $$R_{8}$$ is close to 0, which indicates that *q*_*opt*_ is less than 8 in this iteration. Moreover, we also show the situation when *q* is 5, which is indeed the true number of signals, in Fig. 2A. $$R_{5} $$ is close to 1, suggesting that the *q*_*opt*_ is greater than or equal to 5.

To determine the *q*_*opt*_, we look at $R_{2} , \ldots ,R_{10}$ and locate the significant drop. Specifically, we calculate the first order differences between these values. For instance, we compute $D_{6} = R_{6} - R_{5}$, if $D_{6}$ is found to be the smallest value among all the differences, we conclude that the optimal number of ICs in the first iteration is $q_{opt,1} = 5$. Furthermore, we generate a signal-correlation plot to visually examine if there is a significant drop at the 5th point. A significant drop in the plot suggests $q_{opt,1}$.

In the simulation, we repeat the whole process 10 times, i.e., $Rep = 10$, and record the optimal number of ICs from each iteration as $q_{opt,i} ,\;\;i = 1, \ldots ,10$. Based on previous observations, the performance of CW_ICA, regardless of combining with various ICA methods, become stable in 10 repetitions. Nevertheless, it is essential to adjust the number of repetitions based on the number of input signals to avoid excessive computation time while maintaining accurate outcomes. By examining the 10 signal-correlation lines overlaid on the plot (Fig. 2C), we consistently observe the significant drop occurring at the 5th point from most iterations. Based on this frequent occurrence, we confidently conclude that the optimal number of ICs is $q_{opt} = 5$.

## Assessment of proposed method

Artifacts in the collected scalp EEG signals are inevitable and can affect the subsequent analysis of brain activity. To address this issue, ICA techniques are widely utilized to “clean” scalp EEG signals by filtering out artifacts (e.g., eye movements, cardiac activity, muscle activity, etc.) from brain signals. If too few ICs are used, the resulting brain signal may still contain artifacts, reducing the effectiveness of artifact removal. On the other hand, using an excessive number of ICs can lead to over-separation of the brain signal, potentially causing the loss of important features and information. Therefore, determining the accurate number of source signals is crucial when applying ICA to scalp EEG signals. In this section, we carry out simulations and implement the proposed method on real EEG data to assess the proposed CW_ICA.

### Simulation

Firstly, we assess the performance of the proposed method by applying CW_ICA in conjunction with different ICA methods on simulated EEG signal data. Since the true number of source signals is known in simulation data, the accuracy of CW_ICA along with three determination methods, the DW, ICA-by-Blocks and ICA_corr_y methods, will be compared. We select three widely used ICA methods, namely FastICA, Infomax, and JADE, to combine with each determination method. However, it is important to note that JADE have convergence issues if the preset number of ICs is greater than the number of source signals. Therefore, JADE is only performed on the real data, where the true number of signals is unknown.

#### Simulated data generation

**Figure 3:**
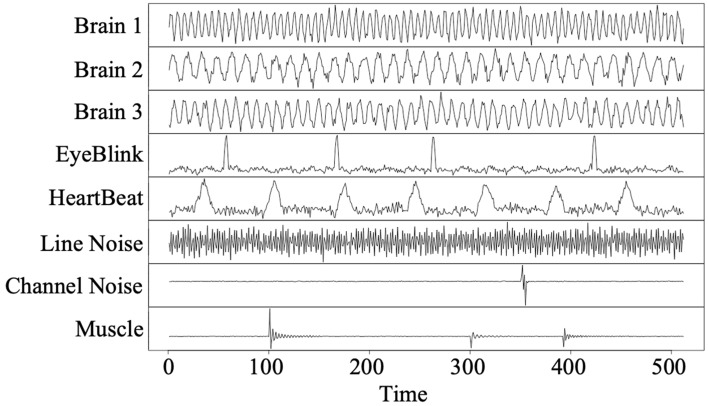
Plots of simulated EEG component signals. The signal length is set as 512.

According to characteristics of EEG components^19^, eight analog EEG source signals (i.e., true $q = 8$), which consist of both periodic and non-periodic signals as shown in Fig. 3, are simulated. Then, $p = 30$ mixed signals, denoted as ${\varvec{X}}$**,** are generated by linearly combining the source signals as ${\varvec{X}} = {\varvec{AS}}$, where ${\varvec{A}}$ is a randomly generated mixing matrix whose elements are normally distributed.

#### Impact of correlation coefficients on determination methods

To assess the impact of different correlation coefficients on two determination methods, specifically ICA-by-Blocks and CW_ICA, we conduct this simulation study. Noting that the true optimal number of ICs is $q_{opt} = 8$ in this simulation.

**Figure 4:**
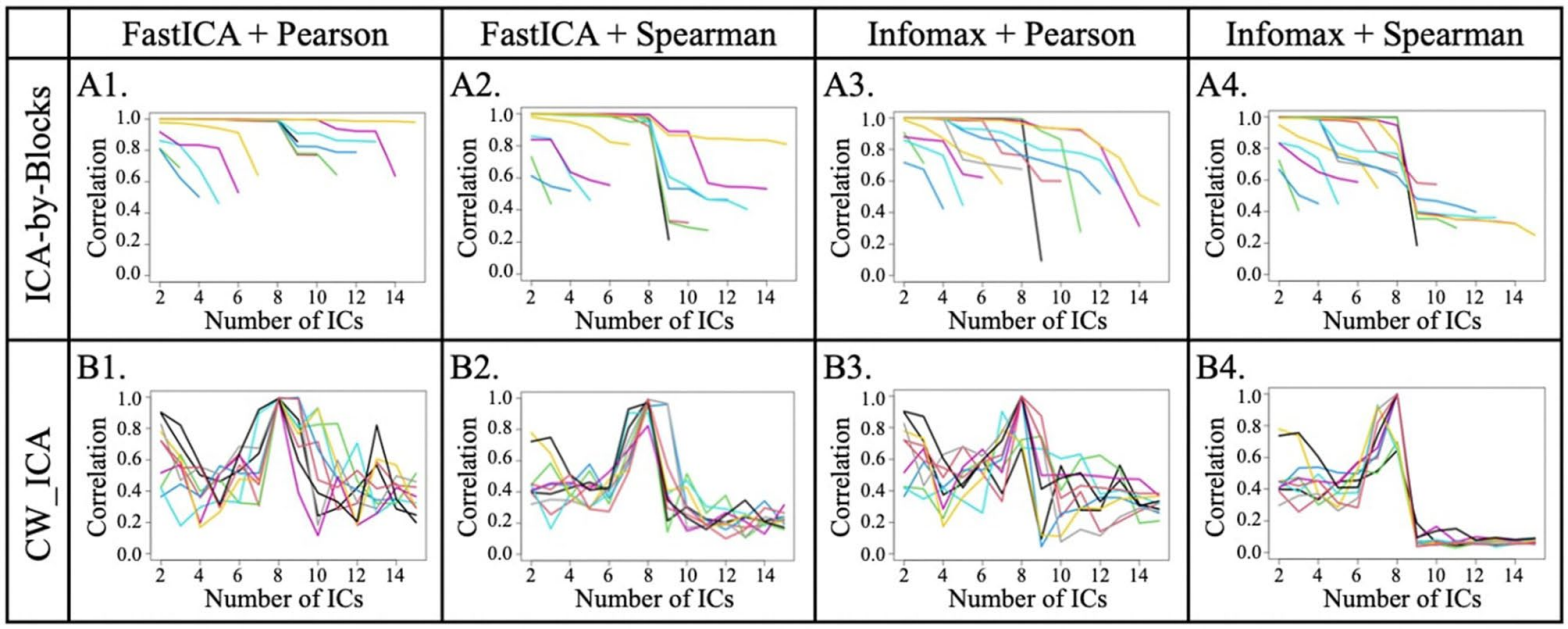
Signal-Correlation plots for (**A**) ICA-by-Blocks and (**B**) CW_ICA methods.

We employ both CW_ICA and ICA-by-block coupled with Pearson and Spearman correlation to determine the optimal number of IC, *q*_*opt*_. Figure 4 shows the signal-correlation plots. For ICA-by-Blocks, when it is coupled with Spearman correlation (Fig. 4A2,A4), the estimated *q*_*opt*_ is 8, which is the same as the true *q*. However, when coupled with Pearson correlation (Fig. 4A1,A3), ICA-by-block determines *q*_*opt*_ being 10, which leads to over-decomposition. This discrepancy suggests that the choice of correlation coefficient greatly influences the determination accuracy of ICA-by-Blocks. On the other hand, CW_ICA, clearly exhibits a sharp drop at the true number of ICs (${\text{ i}}{\text{.e}}{. }\;\;q_{opt} = 8$), which indicates that CW_ICA outperforms ICA-by-Blocks regardless of the type of correlation coefficient employed (Fig. 4B1–B4). Moreover, the result obtained by the Spearman correlation-based CW_ICA provides even much more compelling evidence for accurately identifying the true *q*_*opt*_ (Fig. 4B2,B4). This is because Spearman correlation coefficient captures monotonic relationships that exist among ICs, providing enhanced performance in determining the optimal number of ICs.

#### Accuracy

Since the true number of IC *q* is known in simulation, we can evaluate the accuracy of *q*_*opt*_ obtained by different determination methods including our method and ICA-by-Blocks, DW, and ICA_corr_y. In this simulation, multiple mixed-signal datasets, ${\varvec{X}}$, are generated by repeatedly and randomly generating the mixing matrix, ${\varvec{A}}$, while keeping the original source signal, ${\varvec{S}}$, unchanged. Accuracy in this context refers to the percentage of simulation runs that that a determination method correctly identifies the true number of ICs:

5 $$ Accuracy = \frac{m }{N} \times 100\% $$

where *m* is the number of simulations runs that correctly identify the true number of ICs and $N$ denotes the total number of simulations runs. To estimate the optimal number of ICs, all four determination methods (i.e., CW_ICA, ICA-by-Blocks, DW and ICA_corr_y) are combined with FastICA and Infomax, respectively. Note that source 8 is chosen to be the known source signal required by ICA_corr_y. Limited number of simulations runs (i.e., $N = 5, \;10, \;25$), are carried out because both DW and ICA-by-Blocks methods are graph-based identification methods which are time consuming.

**Figure 5:**
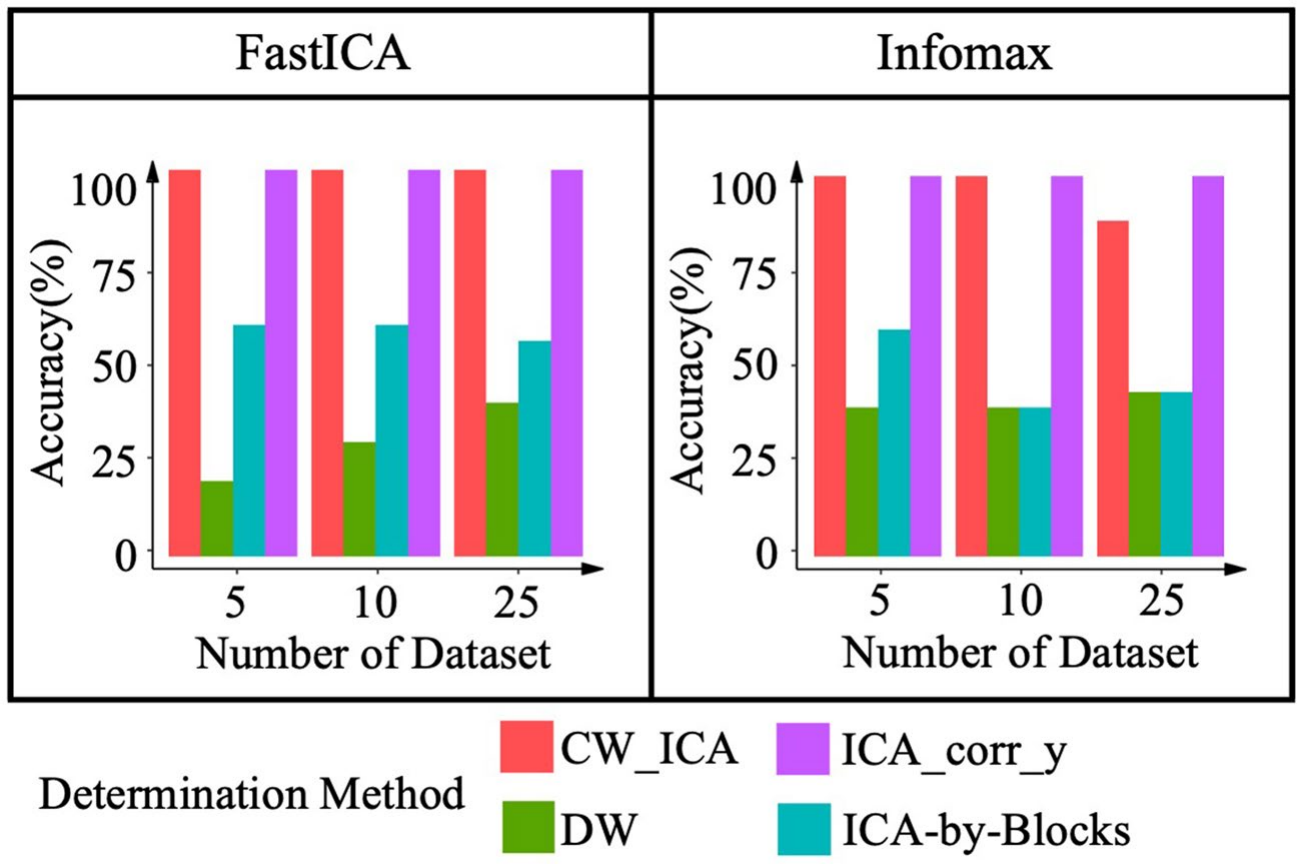
Accuracy of the three determination methods.

As shown in Fig. 5, CW_ICA and ICA_corr_y exhibits the highest accuracy among four determination methods, providing almost 100% accuracy when combined with either FastICA or Infomax. The accuracy of ICA-by-Blocks combined with FastICA is slightly higher than the result when combined with Infomax. However, the DW method performs the worst among the four methods, especially when combined with FastICA.

#### Robustness

To further understand the robustness of CW_ICA and compare with existing determination methods, especially when the observed data has varying characteristics, we simulate datasets, ${\varvec{X}}$, with different properties, such as numbers of mixed signals, signal lengths, signal-to-noise ratio (SNR), and frequency ranges, by changing the parameter setting of simulated source signals, ${\varvec{S}}$, and mixing matrix, ${\varvec{A}}$. In this simulation, the true number of source signals is set to be 5. We compare four determination methods for accurately determining the number of ICs across datasets with diverse characteristics.

**Figure 6:**
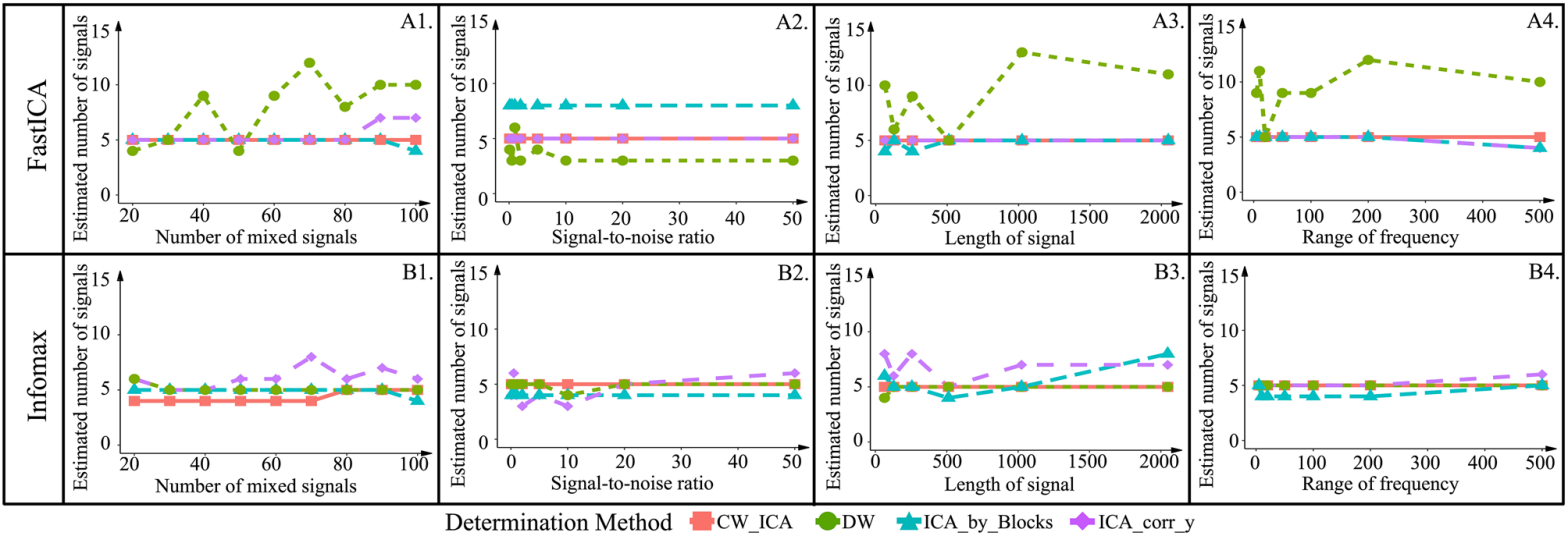
Estimated number of source signals with variety parameters in mixed signals.

Figure 6 illustrates the estimated number of the source signals, *q*_*opt*_, obtained by four methods as we vary the levels of mixed signals, signal lengths, signal-to-noise ratio, and frequency ranges. First, we conclude that DW coupled with FastICA is not suitable, since the Fig. 6A1–A4 show that the results obtained using the DW criteria is inconsistent as the signal parameters change. Second, we observe that if ICA-by-Blocks is used in conjunction with Infomax, it generates inconsistent results in certain cases, for instance as the number of mixed signals increases, or the signal length changes (i.e., Fig. 6B1,B3). Additionally, the results obtained by ICA_corr_y show variations with changes in the characteristics of mixed signals (i.e., Fig. 6B3,B4). Furthermore, when combined with FastICA, ICA_corr_y produces incorrect results under certain conditions (i.e., Fig. 6A2). In comparison, CW_ICA shows more consistent results regardless the characteristic of mixed signals and the ICA methods.

### Scalp EEG data application

Researchers recruited a total of 19 adults (7 females, 12 males) from the University of Arizona in this study^20,21^. Participants were asked to monitor the distances travelled: short (100 virtual meters) vs long (200 virtual meters) distances while navigating in the virtual reality. Each task was repeated 24 trials, and each trial lasted 5.656 s. Participants walked freely on an omnidirectional treadmill while wearing a wireless scalp EEG cap. The sampling rate was 500 Hz. Details of experiment design can be found in^20,21^.

The raw scalp EEG data collected from this study is used to validate the effectiveness and robustness of the proposed CW_ICA, we also consider two other determination methods (i.e., DW, ICA-by-Blocks), with each coupled with three ICA methods (Fast ICA, Infomax, and JADE). In our analysis, we aim to determine the optimal number of ICs at two levels: subject-wise and channel-wise.

**Figure 7:**
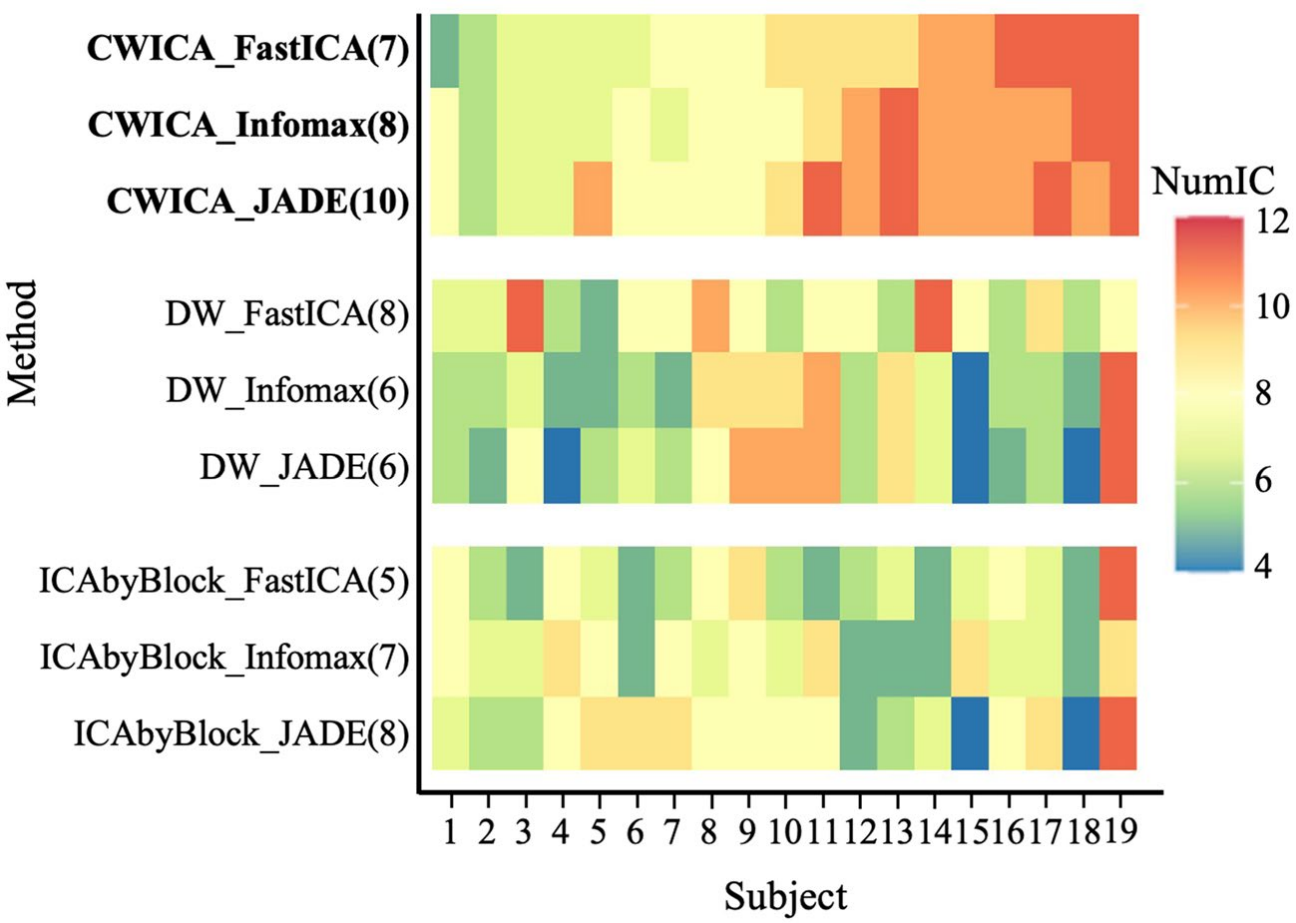
Heatmap of estimated number of ICs. Each column describes the estimated number of ICs by 9 methods from the same subject. Most estimated number of ICs are around 7 to 12, which indicates the number of sources contained in raw EEG signals. The value in parentheses represents the most frequency result of each method.

**Figure 8:**
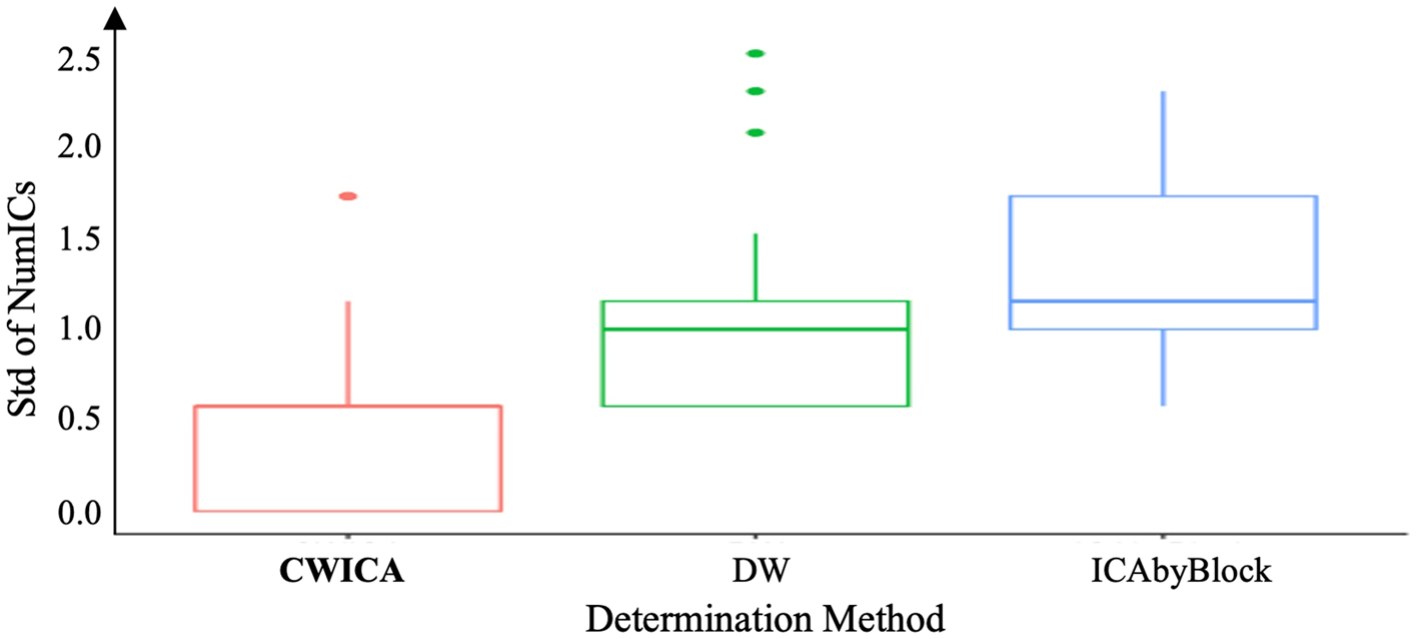
Precision comparison of the estimated number of ICs for three determination methods for every individual.

First, the optimal number of ICs is determined for each subject separately, considering mixed signals X with dimensions of 2828 × 48 (*n*) by 64 (*p*). The heatmap in Fig. 7 shows the optimal number of ICs for each subject, obtained using CW_ICA, DW, and ICA-by-Blocks in combination with FastICA, Infomax, and JADE. We observe that for each subject, the resulting ICs determined by the CW_ICA method exhibit more consistency across the three ICA methods, compared to DW and ICA-by-Blocks. The variation of the determined number of ICs, quantified by the standard deviation (Std), is also summarized in Fig. 8. This indicates that the CW_ICA method has the minimal variation when combining with different ICA methods. The optimal IC number obtained by the CW_ICA method for all subjects is around 7–11 which is close to the widely used IC numbers in neuroscience, while DW and ICA-by-Blocks give smaller IC number, around 5–8. This consistent and reliable performance of CW_ICA in determining the optimal number of ICs, irrespective of the specific ICA algorithm used, underscores its robustness and reliability.

**Figure 9:**
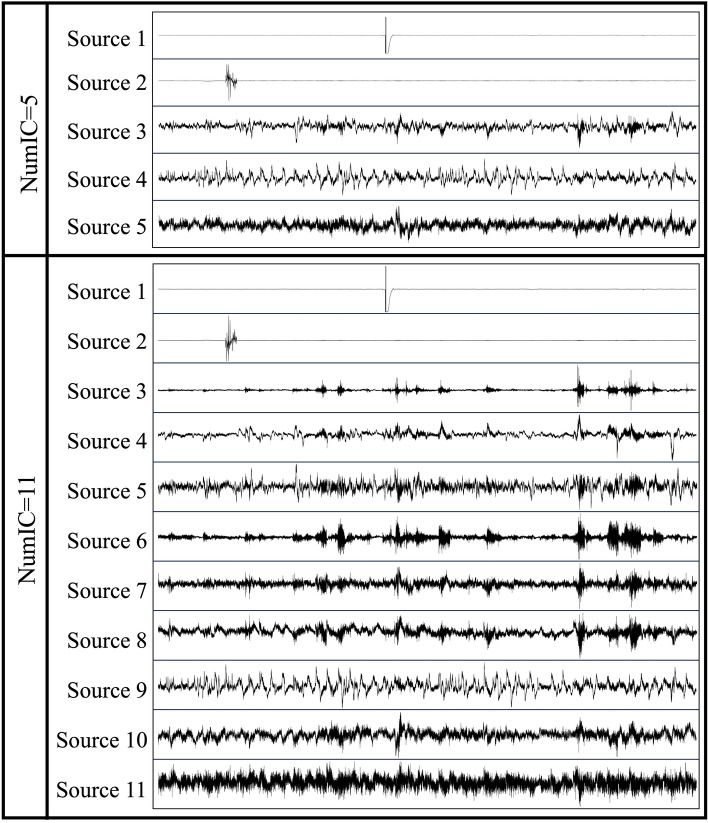
Extracted EEG source signals from subject 18 with different number of ICs.

In real data, there is no ground truth, therefore, we further investigate the corresponding ICs obtained using the optimal IC number determined by three determination methods. Let’s consider subject 18 as an example. The optimal IC number obtained by the CW_ICA method is 11 while the *q*_*opt*_ determined by the other two determination methods is 5. Therefore, we preset the number of IC being 5 and 11 respectively when applying ICA and the obtained ICs are shown in Fig. 9. With the number of ICs being 5, some channel noise can be successfully separated, as evident in IC 1 and 2. However, the remaining three ICs still contain mixed signals and are not fully separated. some channel noise can be separated (e.g., IC 1 and 2). However, the rest three ICs are still mixed signals. With IC number being 11, sources are much better separated, for instance, we obtain the successful separation of channel noise in IC 1 and 2 and, the separation of muscle artifacts in IC 6 and 7.

**Figure 10:**
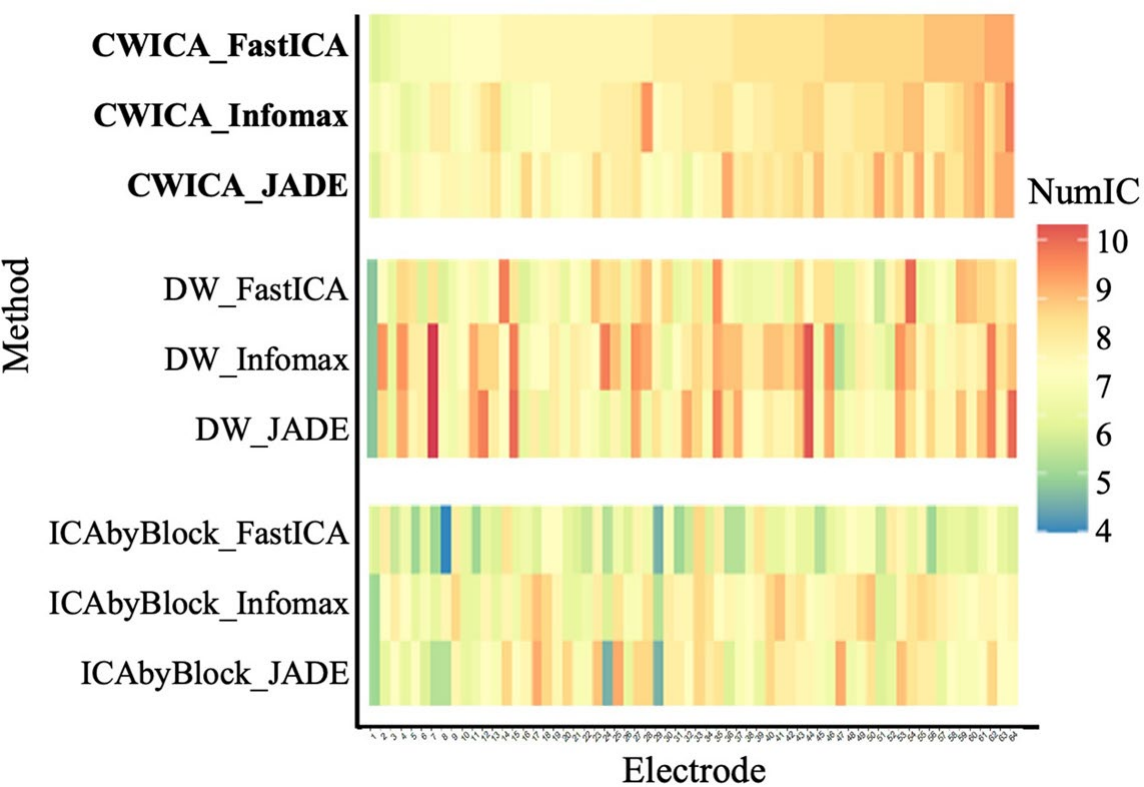
Heatmap of estimated number of ICs. Each column describes the estimated number of ICs by 9 methods at the same electrode.

**Figure 11:**
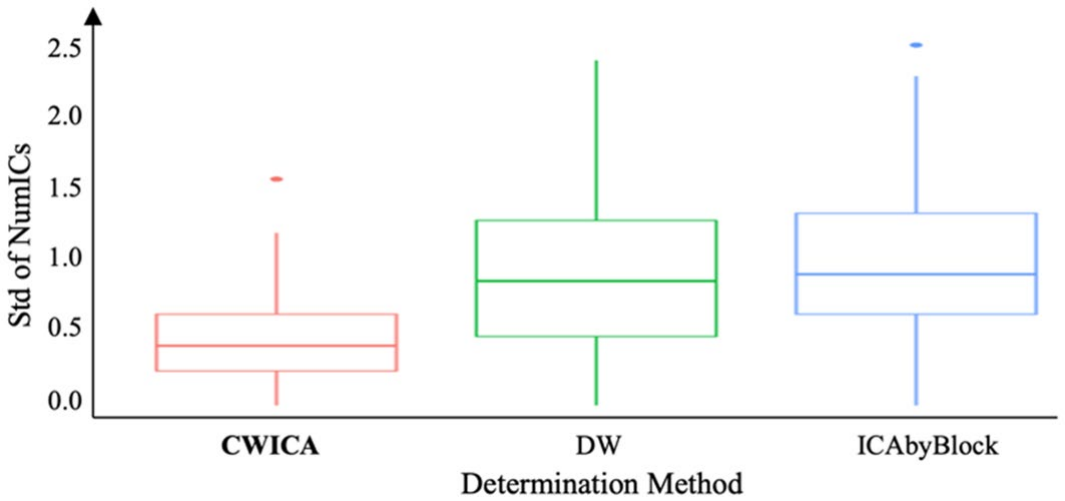
Precision comparison of the estimated number of ICs for three methods of determining the number of ICs for each channel over all 19 individuals.

Besides subject-wise analyses, we also consider channel-wise determination of the optimal IC number. This approach accounts for the possibility that the number of source signals may vary across different brain regions. For example, channels located near the eyes may have a higher number of sources compared to other regions. To determine the channel-wise optimal IC number we implement the nine algorithms on the mixed signals ${\varvec{X}}$**,** with dimensions of 2828 (*n*) by 48 (*p*), for each subject and each channel. The average of obtained optimal IC number across all 19 participants is calculated and summarized in the heatmap given in Fig. 10. When examining the standard deviation (Std) of the determined IC numbers in Fig. 11, it is evident that the CW_ICA method exhibits more consistent outcomes when combined with different ICA methods, even though the optimal IC number may vary across different channels.

**Figure 12:**
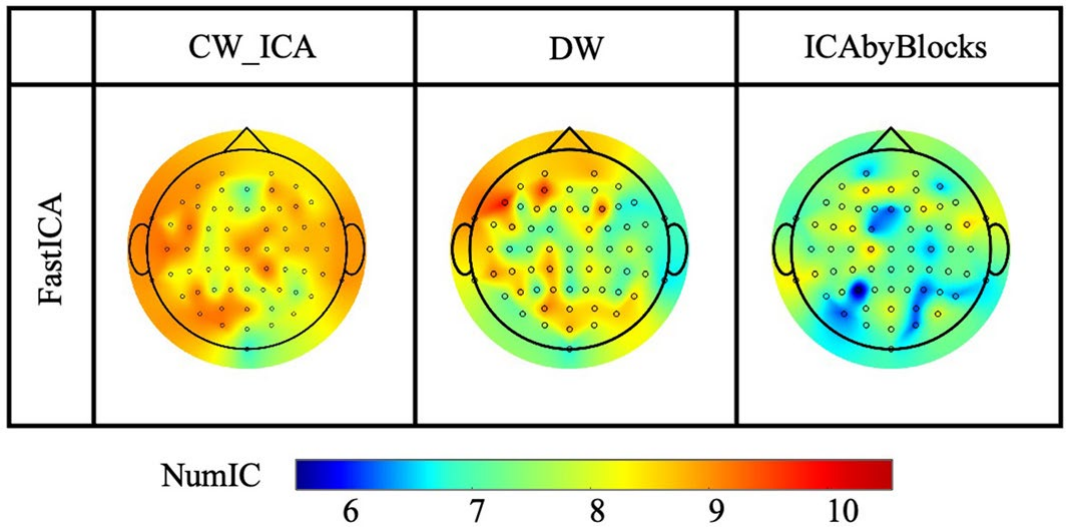
Topographical scalp maps. The number of ICs detected by CW_ICA is relatively larger at electrodes in the temporal and prefrontal cortex than in other brain regions.

Topographical scalp maps (Fig. 12) represent the channel-wise optimal number of ICs for all 19 participants and provide valuable insights into the distribution of the optimal IC numbers across different brain regions. Overall, the optimal IC number obtained by the CW_ICA method is relatively lower in the frontal cortex, indicating potentially less interference from physical artifacts. Whereas a larger number of ICs may be required to effectively separate the brain signals from the artifacts in prefrontal and temporal cortex. This pattern can be attributed to the fact that the prefrontal and temporal cortex regions are near facial muscles and signals are more susceptible to physical artifacts, such as muscle movements and eye blinking. In contrast, the other two determination methods, particularly ICA-by-Blocks, do not show significant variations in the optimal IC numbers across different electrodes.

## Discussion

In this study, we propose a robust method called CW_ICA for determining the optimal number of ICs. Current determination methods visually determine the optimal number of ICs from a plot, they are typically used in conjunction with JADE^9^, and applied to the real data in specific fields. However, there is a lack of information on whether these methods can be effectively combined with other widely used ICA methods, such as FastICA and Infomax, and their robustness in various scenarios. Therefore, to address these gaps and overcome the limitations of existing determination methods, we propose CW_ICA. This method can be combined with multiple ICA methods and automatically determine the optimal number of ICs.

We summarize the significant advantages of the proposed CW_ICA method over existing approaches here. First, its computational cost is significantly lower than the existing methods since it eliminates the need for source signal-related information. Moreover, it relies on a quantitative measurement instead of visual identification. Secondly, the CW_ICA method simplifies the process of determining the optimal number of ICs compared to the ICA-by-Blocks method. Rather than determining the number of blocks in ICA-by-Blocks, CW_ICA divides mixed signals into only two blocks. This simplification eliminates potential challenges in selecting the maximum number of ICs when dealing with a large number of blocks. In addition to the aforementioned advantages, CW_ICA offers several other notable benefits. Firstly, CW_ICA extracts only one value from each column of the off-diagonal matrix, whereas ICA-by-Blocks preserves all values in the off-diagonal matrix. This streamlined approach significantly reduces complexity and computational overhead while maintaining the accuracy and reliability of determining the optimal number of ICs. Secondly, CW_ICA can be coupled with multiple ICA methods, consistently yielding reliable results. Researchers can focus solely on selecting an appropriate ICA method based on the properties of the mixed signal, without concerns about the compatibility of the determination method and the ICA method. Thirdly, CW_ICA is a robust method because it uses rank-based correlation instead of Pearson correlation coefficient as used in ICA-by-Blocks. The rank-based correlation measures the relationship between ICs from different blocks based on ranks, avoiding reliance on assumptions and generating more robust results. Finally, CW_ICA automatically determines the optimal number of ICs. Unlike ICA-by-Blocks and DW, which require visual identification from plots, CW_ICA quantifies the identification process. This quantification allows for automated determination, eliminating the time-consuming manual analysis required by the other methods.

To better illustrate the advantages of CW_ICA, we conduct extensive comparisons using both simulated signals and collected raw EEG signals. First, we compare the performance of two correlation coefficients (Pearson and Spearman correlation coefficients) in combination with ICA-by-Blocks and CW_ICA. The results clearly show that CW_ICA with the Spearman correlation coefficient displays a significant drop at the true number of ICs, 8, providing more accurate determination (Fig. 4). Second, we evaluate the accuracy of CW_ICA by applying it to multiple datasets and comparing it with ICA-by-Blocks, ICA_corr_y and DW. The findings imply that CW_ICA consistently maintains an accuracy rate of nearly 100%, while the performance of the other two methods varies when they are combined with different ICA methods (Fig. 5). Next, to evaluate the robustness of CW_ICA, it is applied to multiple sets of mixed signals with different properties (i.e., length, quantity, signal-to-noise ratio, and range of frequency). We compare the results obtained by CW_ICA with those of ICA-by-Blocks, ICA_corr_y, and DW. The findings indicate that the correct number of ICs is identified by CW_ICA and not affected by changes in the characteristics of the mixed signals (Fig. 6). Further, we compare nine combinations of determination methods (CW_ICA, ICA-by-Blocks, DW) and different ICA methods (FastICA, Infomax, JADE) to determine the optimal number of ICs in raw EEG signals (Figs. 7, 8, 9, 10, 11, 12). Among these combinations, only the proposed CW_ICA provides the same number of ICs across each electrode, demonstrating its compatibility with different ICA algorithms and consistent determination capability. It is worth noting that our methods are adaptable and can be applied to other datasets for the determination of the number of source signals as well.

While CW_ICA demonstrates consistent results when combined with multiple ICA methods, the efficiency of ICA is inherently tied to the selection of specific ICA methods, which are determined based on the characteristics of the signal data. Additionally, the validity of the assumption in ICA that source signals are mutually statistically independent can impact the overall performance of CW_ICA. Moreover, the computational complexity escalates with an increase in the number of mixed signals and signal length, as the study exclusively focuses on classical ICA methods. Furthermore, the study imposes limitations on the choice of ICA methods by assuming that the number of source signals is less than or equal to half the number of mixed signals (i.e., $q \le \frac{p}{2}$). To broaden the scope, future investigations will explore other over-determined ICA methods, aiming to unleash the potential for a greater number of source signals. Additionally, CW_ICA can be expanded to functional CW_ICA, considering the time-dependence of data, thereby mitigating the impact caused by the length and pattern of the signal.

## Conclusion

The proposed CW_ICA method addresses limitations in current determination methods by introducing a quantitative measurement and a block splitting approach to reduce computational complexity. By focusing on the smallest column-wise maximum absolute value, CW_ICA offers a versatile solution that can be seamlessly integrated with various ICA methods. Moreover, it leverages the robustness of the Spearman correlation coefficient, which leads to reliable and consistent results in determining the optimal number of ICs automatically. To evaluate the performance of CW_ICA, it is compared with existing determination methods in combination with multiple ICA methods using extensive simulated data and real raw EEG signals. In conclusion, the proposed CW_ICA method offers a versatile and robust approach for automatically determining the number of ICs in signal analysis. Its compatibility with multiple ICA methods, reduction in computational complexity, utilization of Spearman correlation coefficient, and strong performance in comparative evaluations make it a valuable tool for researchers in various fields.

## Data Availability

The scalp EEG datasets are available at https://osf.io/3vxkn/. Written informed consent was obtained from participants and study was approved by the Institutional Review Board at the University of Arizona and is conducted in accordance with relevant guidelines and regulations. Additional simulated datasets and code are available at https://github.com/yzy0080/CW_ICA.git.
